# Editorial and Miscellaneous

**Published:** 1873-11

**Authors:** 


### PART III.

### EDITORIAL AND MISCELLANEOUS.

*To our Subscribers.*

☞ LOOK OUT FOR THE RED CROSS !!

*Our Sincere Thanks—*

Are due to the large number of subscribers who have, in response to our appeal for money, so promptly forwarded their dues. This we felt sure they would do when made aware of our wants in the premises. As we are obliged to pay our publisher cash for every number as soon as it is issued, we know all our friends appreciate the situation and rejoice to lend us a helping hand. A few subscribers are still in arrears. We know they, too, will appreciate our difficulties, (with a money panic to increase them,) and hope very soon to have the pleasure of " squaring" all subscriptions on our books. It is also necessary to make full settlement for the year in order to commence the coming year unembarrassed. We trust all who owe us will remember this, and oblige us by forwarding their dues without delay.

☞ We can furnish a few new subscribers with the back numbers, if they wish to have this volume complete. It will be invaluable as a book of reference.

☞ All subscribers, and others, will please write their names, P. O., County and State plainly. ☞ Remember, this is now required by the Postmaster General.

☞ Should any mistake occur either in name or post - office, or should the RECORD fail to reach its destination, please inform the editors, who will have matters righted without delay.

☞ We invite our readers and friends every where to send us communications. Overhaul your case-books and send us your experience. Our columns are open to all.

☞ We will take the liberty to cull from private letters such extracts as may be deemed of special importance in promoting the interests of the profession.

☞ Dr. W. T. Taylor, of Kentucky, writes: "I will be one of two hundred who will send five new subscribers, each accompanied by the cash, between this and Christmas."

☞ The above proposition we place before our readers, and pledge ourselves (if two hundred of our subscribers will agree to pledge themselves to send us five new subscribers each by the first of January) to enlarge the size of the journal without additional cost, and to give a premium (in books, instruments or anything else desired) to the value of seventy-five dollars to the person getting the largest number of names over five, fifty dollars to the next, and twenty-five to the next. If fifty will send us five new subscribers, the one that sends the largest number over five shall have a premium of his own choosing worth twenty-five dollars. Go to work, friends; you have from now until the first of January to get up your list of names for this volume and the next, counting from the issuing of the August number, or the first of September.

☞ In addition to the premiums already offered, each new subscriber will be entitled to Wood's Household Magazine and the superb chromo—the Yosemite Valley, 14x20—for $3.30. So, for a small sum, the RECORD, a fine LITERARY MONTHLY and a *magnificent chromo* may be had.

Any one sending us three subscribers ($7.50) will be entitled to POWELL'S FORMULARY, or WOOD'S HOUSEHOLD MAGAZINE and CHROMO, gratis.

*Powell's Pocket Formulary.*

On another page we present the contents of the above named *Formulary*, which will show, at a glance, its character, scope and design. This is done because we have offered the *Formulary* as one of the premiums to subscribers. All persons who have sent us two (2) subscribers, by sending one more will be entitled to a copy gratis, when we are notified of the fact. All who will send us, from the September number, two (2) new subscribers, will be entitled to a copy free.

Communications are solicited from all professional gentlemen every where. Send your articles along. Friends, *write!* WRITE!! WRITE!!!

*Dr. Thomas S. Powell vs. Dr. Jesse Boring, et. al. Libel, etc.*

This suit brought by Dr. Powell against Drs. Boring, Willis F. Westmoreland, John G. Westmoreland, and others, for Libel, with the facts of which many of our readers are familiar, was brought to a close in the Superior Court of Fulton County, Ga., on the 16th inst., by a total withdrawal, on the part of the defendants, of every injurious and offensive publication to the plaintiff, (Dr. Powell,) ; and as Dr. Powell in bringing the suit was only moved to do so for the purpose of publicly vindicating his character, he of course could ask no more, and in a spirit of conciliation consented that the case should be settled, and dismissed, on the mutual withdrawal of all offensive publications. The case now being settled in the courts, we hope that our numerous readers will dismiss it from their minds, and continue to aid us in our efforts to advance the objects advocated and discussed in the SOUTHERN MEDICAL RECORD.

*Dr. J. Q. A. Hudson, of Ohio.*

This gentleman, in a private letter, from which we take the liberty of "clipping" a paragraph, says:

"The many practical items in the RECORD will make it of immense utility to the practicing physician, if the index is so full that he can find easily whatever he may wish to refer to."

The compliment contained in the above is fully appreciated. We desire the profession to know, when subscribing for the RECORD, they will get all they bargain for. We desire to make and to some extent hope we have already made, a practical journal supplying the every day wants of the busy practitioner. Every volume, so far, has been copiously indexed, so that every item can be readily found. When the present volume is completed, our friend will find an index which will enable him to place his hand upon any item at any moment. We will say in this connection, that complete volumes of the present number, bound and indexed, can be furnished to a limited number, at the close of the year, which will be forwarded at cost. We would be glad to have our subscribers dispose of 50 volumes to their friends. Each volume will cost about $4.00. Will they not aid us ?

*Dr. S. S. Satchwell.*

This gentleman delivered, recently, an address of great merit before the Eastern Medical Association, which we will publish in our December number.

*The Southern Medical Record.*

The first volume of the RECORD ends with the next (December) issue. We can furnish from fifty to seventy-five copies complete, either bound or unbound. If bound, it will cost about $4.00. Will not our friends secure us fifty new subscribers within the next twenty days? We believe they can. We are authorized to say that Dr. T. Curtis Smith will secure us five new subscribers. Remember, those who send us two new subscribers will receive a copy of "Powell's Pocket Formulary." This volume of the RECORD, well indexed and bound, will make a book for every day reference, that is seldom seen. It will be invaluable. We desire our friends to have in that form a book of reference that they may consult daily, and with little trouble. New features which we are preparing to make for next year will, we think, greatly increase the value of the journal, and which will be stated in the next issue.

*Advertisements.*

We call attention to our advertisement department. Owing to a mistake of the printer, the advertisement of Henry K. Wampole & Co. was *sandwitched* between the pages containing our large advertisement of Alex. Browne & Co. The intelligent reader will see and appreciate our apology to these gentlemen, while we take the opportunity of saying they will find them first-class drug houses in every particular.

Dr. E. Cutter & Brother offer vaccine virus from kine to the profession through our advertising pages. As cases of small-pox are constantly occurring in every part of the country, it would be well for physicians to remember they can secure genuine, non-humanized vaccine virus by applying to these gentlemen. Prices are given in the advertisement, which please see.

*Communications*—

Have been received from the following gentlemen, and will be published in our next number: J. Q. A. Hudson, M. D., of Ohio; A. K. Kilpatrick, M. D., of Texas; J. W. M. Shattuck, of Miss.; and Thos. S. Mitchell, M. D., of Georgia.

Our friends, everywhere, are cordially invited to make themselves known to our readers, and the medical world, through our pages.—The *audience* of the "Record" is a large and increasing one. Few medical journals in this country enjoy a larger circulation, and we feel sure those that do are published North of Mason & Dixon's line. Please bear this in mind, and forward your communications. We will be pleased to receive the additional communication promised by Dr. Hudson.

*Our Advertising Department.*

We have always believed, and experience has only strengthened our belief, that a department of select advertisements is an indispensable feature of a medical journal. The manufacturer of pure chemicals and drugs and surgical instruments should have a direct medium for communicating knowledge of his business to the medical profession; and on the other hand, the members of the profession should be kept posted as to where they can get what they may need in their practice, as well as in all the improvements in the manufacturing of instruments, appliances, chemicals, drugs, etc. In view of these facts, we have determined to change our advertising system, in order to make it still more effective and valuable, both to advertising and our readers.

We shall, during the coming year, devote only six pages to our advertising department—two pages on the cover, and four at the close of the journal. And in this respect we beg leave to say that, though the journal may not have as many subscribers as a few medical journals in the North, still, its influence is second to none circulating, as it does, over an immense territory, and penetrating to remote sections North and South. It is, therefore, unexcelled as an advertising medium, which fact all interested should not fail to remember.

Our rates for the ensuing year will be as follows:

| | |
|---|---|
| For one page per annum | $100 00 |
| For half page per annum | 60 00 |
| For quarter page per annum | 30 00 |
| For one page one insertion | 15 00 |
| For half page one insertion | 10 00 |
| For quarter page one insertion | 7 00 |

For anything less than the above, fifty cents per line each insertion.

Advertisers in every case will have the privilege of *changing* their advertisements *each issue,* if desired, thus giving them an opportunity each month to bring before our readers, something new and of great interest. Advertisements will be attractively displayed, and fully noticed in the editorial columns.

*Proceedings of Societies.*

While our readers object to our filling our pages with uninteresting details of Medical Societies, yet we would be pleased to have our friends send us the interesting and instructive portions of the proceedings of their societies for publication. Friends, let us hear from you.

*Electrical Instruments.*

We are authorized to state that Messrs. Redwine & Fox, the "Live Drug Store" of Atlanta, have for sale several sizes of the Electrical Instruments made by the Galvano Faradic Manufacturing Company, New York. Druggists and Physicians have now an excellent opportunity to get these instruments as cheap as they can be bought in New York. Address, at once, Messrs. Redwine & Fox, Atlanta, Ga.

*Eastern Medical Association of North Carolina.*

The "Old North State" has taken another step for the promotion of Medical Science in the organization of the above named Society. It is composed mainly of young medical gentlemen, and promises much for the medical future of North Carolina. The following are the officers for the ensuing year: Dr. Charles Duffy, of Newbern, President; Dr. W. T. Ennet, of New Hanover, Dr. L. A. Stith, of Wilson, Dr. Rountree, of Green, and Dr. Kirby, were elected Vice-Presidents; Dr. H. O. Hyatt, of Lenoir, Secretary, and Dr. Hadley, of La Grange, Treasurer; Dr. J. F. Miller, of Goldsboro, Orator. The Executive Committee consists of Drs. S. S. Satchwell, W. T. Ennett and H. W. Faison.

We wish the Association great prosperity, and hold the pages of the RECORD open for communications read before it.

In this connection, it affords us a real pleasure to state that the RECORD has a larger circulation in North Carolina, probably, than any other State. Without consultation with us or one another, the medical men of that State have virtually made it their organ. We thank our many friends of North Carolina for the kind interest they have manifested in extending the circulation of our journal, and hope it will ever prove an acceptable medium for them.

*The Medical Society of Virginia.*

The fourth annual meeting of this Society was held at Norfolk, Va., on the 13th ult. The Society was called to order by the President, Dr. Harvey Black.

After the calling of the roll, the President introduced Dr. Samuel Selden, of Norfolk, who addressed the Society in a happy and most tasteful speech of about fifteen minutes' length, in which he extended a cordial welcome in behalf of the profession in Norfolk, to the visiting delegates from various sections of the State, and a special welcome to those who were present from North Carolina. Dr. Selden's speech was listened to with the most profound attention and earnestly applauded.

Upon the conclusion of Dr. Selden's speech, Dr. Robert S. Hamilton, of Staunton, was introduced as the orator of the evening, and he proceeded to address the Society, taking for his subject: "The Relation of Medicine to Society and some of their Reciprocal Obligations." He spoke for over an hour in a most interesting manner, showing himself the real type of a true physician and orator, who relies upon the integrity and fidelity of the profession for its success.

At 10 P. M. Dr. Hamilton closed his admirable address, and the President stated the next business in order to be a report from the Committee on Membership. The Secretary read the report of the Committee, recommending a large number of medical gentlemen from all sections of the State for connection with the Society. The report was received by a unanimous vote.

The Secretary's report was then read and the present membership given as 295—three members having died during the year, viz: Drs. R. S. J. Peebles, Thomas P. Perkins and Albert Snead.

Dr. Thoms P. Atkinson read an address replying to certain criticisms made by the Boston *Medical and Surgical Journal* on a certain paper which he submitted to the Society at its third annual session on the subject of "Anatomical, Physiological and Pathological differences between the white and black races, and on the modification of the treatment of diseases of the latter rendered necessary thereby."

Dr. Hunter Maguire, of Richmond, read a most interesting essay on "Wounds Penetrating the Abdominal cavity and the Performance of Ovariotomy" giving particular instances coming under his personal treatment.

A paper was read by the Secretary on Malarious Diseases, by Dr. Wm. A. Gillespie, of Louisa county.

At this stage of the proceedings the President delivered the annual address. He presented two subjects for the consideration of his fellows. First. What has for a long time been a topic of conversation among medical men throughout the State, exciting their various criticisms, viz: The number of what are known by the profession as "Irregular Practitioners," men varying in their professional capacity from the uneducated, superstitious doctor, to the man of considerable medical attainments, but who is still without the professional qualifications necessary to entitle him to the confidence of his patients and the recognition of members of this Society, and of other skillful physicians practicing in the same community with him.

This subject was held by Dr. Black to be one of very great importance, and eminently proper to be discussed in this Convention. Some measures should be taken to secure the passage of a bill by the Legislature, requiring a regularly prescribed examination for every medical man desiring to practice in the State. He held it to be as necessary that an examination be imposed upon men of this profession as upon those entering the profession of law.

The second subject presented was the necessity of invoking some favorable action of the Legislature to prevent the sale of adulterated medicines. He urged this matter upon the members, and sustained his views with much earnestness and ability.

Dr. W. C. Dabney, of Charlottesville, read a paper on "Nitrite of Amyl as an Antidote for Chloroform."

Dr. A. M. Fauntleroy, of Staunton, Va., read a paper on "The Use of Chloroform in Obstetrical Practice." Upon the conclusion of this essay, Dr. J. H. Claiborne, of Petersburg, spoke at considerable length upon the same subject. Several gentlemen participated in the discussion, which was continued for some time.

Dr. Edwards submitted a paper by Dr. Wm. J. Jones, of Amherst county, Va., on certain treatments in a particular case coming under his practice.

A paper was submitted on "Saccharated Pepsin," by Dr. C. Wesley Thomas, of Norfolk.

On motion of Dr. Wm. L. Broaddus, the Society went into the discussion of the pathology and treatment of Cerebro-Spinal Meningitis, that being the subject selected for this session.

Dr. T. J. Riddell read an interesting paper upon, and expressed some opinions in regard to several cases of this disease coming under his practice. Drs. W. W. Parker, L. B. Edwards and O. B. Jenks participated in the discussion.

The following officers were elected for the ensuing year:

President—Dr. Alfred G. Tebault, of London Bridge, Princess Anne.

First Vice-President—Dr. S. C. Gleaves, of Wytheville.

Second Vice-President—Dr. L. S. Joynes, of Richmond.

Third Vice-President—Dr. Benj. Blackford, of Lynchburg.

Fourth Vice-President—Dr. O. B. Jenks, of Culpepper.

Fifth Vice-President—Dr. R. S. Hamilton, of Staunton.

Sixth Vice-President—Dr. M. N. Lewis, of Alexandria.

Recording Secretary—Dr. L. B. Edwards, of Richmond.

Corresponding Secretary—Dr. F. D. Cunningham, of Richmond.

Treasurer—Dr. L. B. Edwards, of Richmond, who, it is recommended, will assume the combined duties of Recording Secretary and Treasurer.

Committee on Nominations—J. B. McCaw, T. J. Riddell and M. M. Walker of Richmond; Jas. C. White, of Farmville, and W. R. Weiseger, of Manchester.

Executive Committee—W. W. Parker, of Richmond; John S. Apperson, of Smythe county, seven mile Ford; Wm. D. Hooper, of Liberty, Va.; and W. S. McChesney, of Staunton, Va.

Committee on Publication—J. S. Wellford, of Richmond; J. L. Cabell, of University of Virginia; M. C. Christian, of Lynchburg; and L. B. Edwards, *ex officio* member.

Dr. M. P. Christian, of Lynchburg, was chosen annual orator. The next session of the Society will be held at Abingdon, Va.

Our thanks are due our associate, Dr. Robert B. Tunstall, of Norfolk, for copies of the Norfolk Daily Landmark, from which we have condensed the above.

